# EXAMINATION OF STAFFING STABILITY IN LONG-TERM CARE

**DOI:** 10.1093/geroni/igad104.3313

**Published:** 2023-12-21

**Authors:** Winnie Sun, Jen Calver, Farzana Rahman

**Affiliations:** Ontario Tech University, Oshawa, Ontario, Canada; Ontario Tech University, Oshawa, Ontario, Canada; Ontario Tech University, Oshawa, Ontario, Canada

## Abstract

**Background:**

Long-term care (LTC) homes in Ontario, Canada face challenges of recruiting and retaining qualified staff prepared to address resident care needs.

**Objectives:**

To gain knowledge of the barriers and facilitators associated with recruiting and retaining nurses (RPNs/RNs) and personal support workers (PSW) in LTC.

**Method:**

Online survey questionnaires were distributed to staff working in a municipal home, and to PSW and nursing students. Staff participants (n=93) ranked how they perceived their work in LTC during COVID-19 to inform staffing stability efforts, and were invited to elaborate further on their responses using open-ended questionnaire. Nursing student participants (n=11) ranked how they perceived working in LTC.

**Results:**

Findings revealed that staff participants (57%) reported intentions to remain in their LTC employment, leaving about 43% of the workforce at risk of leaving. Four themes emerged from this study: (1) Embracing resident centred care as the top priority; (2) Rebuilding a health workplace through enhanced leadership and organizational support; (3) Promoting quality of care through open communication and professional development opportunities; and (4) Transforming work scheduling policies and staffing practices to support workforce retention.

**Conclusion:**

Senior leaders and LTC organizations play a critical role in refocusing staffing stability efforts. To help make LTC a workplace of choice, major changes must be considered to include greater visibility and presence of leadership, ongoing training and education, and revisit scheduling policies and practice with input from part-time and casual staff.

